# Prospects for liquid biopsy approaches in lymphomas

**DOI:** 10.1080/10428194.2024.2389210

**Published:** 2024-08-10

**Authors:** Esraa Jamal, Edward Poynton, Mohamed Elbogdady, Sameh Shamaa, Jessica Okosun

**Affiliations:** a Centre of Haemato-Oncology, Barts Cancer Institute, Queen Mary University of London, London, UK; b Clinical Haematology Unit, Oncology Center, Mansoura University, Mansoura, Egypt

**Keywords:** Lymphomas, circulating tumor DNA (ctDNA), liquid biopsy, next generation sequencing (NGS)

## Abstract

Analytes within liquid biopsies have emerged as promising alternatives to traditional tissue biopsies for various malignancies, including lymphomas. This review explores the clinical applications of one such liquid biopsy analyte, circulating tumor DNA (ctDNA) in different types of lymphoma, focusing on its role in diagnosis, disease monitoring, and relapse detection. Advancements in next-generation sequencing (NGS) and machine learning have enhanced ctDNA analysis, offering a multi-omic approach to understanding tumor genetics. In lymphoma, ctDNA provides insights into tumor heterogeneity, aids in genetic profiling, and predicts treatment response. Recent studies demonstrate the prognostic value of ctDNA and its potential to improve patient outcomes by facilitating early disease detection and personalized treatment strategies Despite these advancements, challenges remain in optimizing sample collection, processing, assay sensitivity, and overall consensus workflows in order to facilitate integration into routine clinical practice.

## Introduction

Over the last few decades, techniques for probing liquid biopsy analytes have emerged in research as a viable alternative to traditional tissue biopsies for different types of malignancies. The term liquid biopsy refers to the minimally invasive collection of a bodily fluid sample such as blood, urine, and cerebrospinal fluid and its subsequent analysis. There is a range of possible analytes within liquid biopsies, approaches originated with the analysis of circulating tumor cells (CTCs) in peripheral blood samples but have expanded to encompass circulating tumor DNA (ctDNA), circulating cell-free RNA (cfRNA), extracellular vesicles (EVs), and tumor-educated platelets (TEPs). Of these, the majority of studies to date have focused on the analysis of ctDNA, the proportion of total cell-free DNA (cfDNA) which is tumor-derived [1].

Cell-free DNA (cfDNA) was discovered in human blood in 1948, with the presence of fetal-derived cfDNA in maternal plasma noted in 1977. cfDNA concentrations in patients with metastatic tumors are significantly higher than in healthy individuals due to a significant proportion of tumor-derived cfDNA, so-called, ctDNA. Leveraging advances in next-generation sequencing (NGS) technology has supported the progression of ctDNA analysis from polymerase chain reaction (PCR)-based single site mutation profiling for recurrent point mutations, to multi-omic analyses encompassing, mutation and copy number profiling, methylation, fragmentomics, and more recently, machine learning, and artificial intelligence (AI) [2].

Lymphomas (comprising both Hodgkins’ and Non-Hodgkin Lymphomas (NHL)) are the most common hematological malignancy. There are over 60 different subtypes of NHL with an estimated 544,000 new cases and 260,000 deaths, based on 2020 data from the Global Cancer Observatory (GLOBOCAN), National Cancer Institute (NCI), Surveillance, Epidemiology, and End Results (SEER), and World Health Organization (WHO)- databases [3]. Despite good overall response rates in NHL, a significant proportion of patients will experience disease relapse or be refractory to initial therapy.

Both diagnosis and relapse disease assessment have traditionally been assessed by tissue biopsy, the gold standard for disease determination in lymphoma. However, tissue biopsy can be challenging to obtain due to challenging anatomical locations of disease, patient unsuitability for invasive procedures, and lymphoma-associated morbidity. Recent studies have demonstrated both spatial and temporal genetic heterogeneity in NHLs such as diffuse large B-cell lymphoma (DLBCL), mantle cell lymphoma (MCL), and follicular lymphoma (FL) and highlighting the inadequacy of a single spatiotemporal lymphoma biopsy in assessing the entire burden of lymphoma which can involve multiple nodal sites, solid organ disease, as well as secondary spread to immune privileged sites such as the brain and spinal cord [4–7]. The limitations of single-site tissue biopsy are notable in certain clinical scenarios, for example, determining the presence of transformed disease in patients who have been treated for low-grade lymphomas.

There have been considerable advances in the understanding of the molecular basis of lymphoma aiding lymphoma diagnoses and raising the consideration of genomic-based lymphoma classification systems [8,9]. These advances and reclassification of lymphomas highlight the importance of genetic assays in lymphoma diagnostics and disease tracking. As a result, there has been, and remains, considerable interest in multi-omic analysis of liquid biopsy samples in lymphoma.

This review highlights the growing studies evaluating ctDNA-based assays in NHLs and explores their potential clinical implications. We review recent updates in the use of ctDNA-based liquid biopsy tools for the diagnosis, disease monitoring, and relapse detection of lymphoma. We discuss the clinical implications of ctDNA in different types of lymphomas and summarize the updates in liquid biopsy technologies, prospects for using ctDNA, and remaining barriers to their incorporation in routine clinical practice.

## Pre-analytical and methodological considerations

The concentration of cfDNA in lymphoma patients is higher than in healthy individuals and ranges from 5-100 ng/ml with a median of ∼25 ng/ml (7500 hGE/ml). In patients with lymphoma, ctDNA accounts for 5% of plasma-derived cfDNA in diagnostic samples, with higher concentrations of ctDNA being associated with larger tumor volumes and high-grade disease. Plasma cfDNA is rapidly cleared from the circulation and its short half-life has implications for collection and analysis approaches [10].

Isolating and stabilizing this small fraction of ctDNA is challenging, requiring optimization of sampling techniques, collection tubes, post-collection sample processing, and storage in order to maximize ctDNA recovery. Clarity on the required cfDNA input quantities for detection assays and the establishment of pre-analytical standard operating procedures remain necessary hurdles for the incorporation of ctDNA assays into routine clinical practice. For blood draws, plasma is preferred to serum due to lower ctDNA fractions in the latter and at least 10 ml of blood drawn to maximize the quantity of ctDNA obtained [10].

Due to the low frequency of potential genetic variants of interest, optimizing assay sensitivity is of paramount importance in the development of ctDNA assays. A key obstacle to obtaining high levels of sensitivity is the inherent error rate intrinsic to next-generation sequencing technologies owing to PCR-based errors introduced during genomic library preparation and on-sequencer detection errors during library sequencing [11]. With improvements in library preparation (such as the incorporation of molecular barcoding, sequencing technologies and bioinformatic digital error suppression), this error rate can be reduced to approximately 0.01%. However, this sensitivity remains orders of magnitude below what is required to accurately detect minimal residual disease [12,13].

Analysis of ctDNA genomic profiling data without reference to paired data from a tumor biopsy sample (a tumor agnostic approach) has a number of challenges including the detection of false positive variants due to the inability to use a tumor-specific “pre-selector” panel of known variants to track. Some of these limitations can be ameliorated through tandem analysis of ctDNA and paired tumor DNA from a tissue biopsy (a tumor-informed approach). This allows for the identification of false negative variants in ctDNA and is better suited for broader minimal residual disease monitoring and the detection of disease recurrence. Even in tumor-informed analysis, genetic variants associated with clonal hematopoiesis (CH) remain a source of false positive biological noise in cfDNA. CH represents clonal expansions in blood cells driven by somatic mutations with hematopoietic stem cells. Simultaneous sequencing of cfDNA with matched leucocyte-derived DNA commonly implemented to filter and remove CH-variants [14].

PCR-based techniques such as allele-specific PCR and droplet digital PCR (ddPCR) have been used extensively in ctDNA analysis. DdPCR in particular has a high limit of detection of about 0.01% but is dependent on individual probe sets for each target limiting its utility in complex multiplex assessment [15]. NGS-based techniques have broadened ctDNA assays and include the Safe-Sequencing system (Safe-SeqS), cancer personalized profiling by deep sequencing (CAPP-seq), integrated digital error suppression enhanced CAPP-seq (iDES-enhanced CAPP-seq), clono-SEQ and phased variant enrichment and detection sequencing (PhasED-seq) [16–19].

ClonoSEQ (Adaptive Biotech) relies on the use of multiplex primers targeting the immunoglobulin heavy and light chains together with NGS to identify and quantify tumor-specific clonotypic rearrangements. CAPP-seq detects tumor-specific mutations in ctDNA using a selector probe set designed for the specific tumor type of interest and suppresses technical errors through the use of a unique barcoding strategy together with a downstream bioinformatic algorithm that eliminates sequencing errors and stereotypic background noise allowing ctDNA variant detection down to ∼0.002%. Variant detections sensitivity has been further improved through the implementation of phased variant enrichment and detection sequencing (PhasED-seq), which tracks two or more phased variants on the same strand of DNA molecule. This method lowers both the technical and background signal-to-noise ratio enabling ctDNA monitoring down to a detection limit of ∼0.00005% [19].

## Clinical applications of ctDNA in lymphoma

### ctDNA studies in DLBCL

The earliest studies examining ctDNA in NHLs began with DLBCL, the most common lymphoma worldwide, and utilized immunoglobulin high-throughput sequencing (IgHTS, now clonoSEQ) to track patients’ tumor-specific Ig clonotypes throughout their treatment and during remission. These studies demonstrated the high rates of detection of clonotypic ctDNA in plasma, that levels correlated with imaging modality assessment of disease burden and that ctDNA has prognostic value in identifying disease relapses [19,20]. Later studies matured to focus on disease monitoring using broader targeted gene panels specific for DLBCL and underscore its role as a prognostic biomarker at baseline, at interim time points and end of treatment. Table 1 shows the key studies demonstrating the applications of ctDNA in DLBCL and other types of lymphoma.

**Table 1.** Key studies demonstrating the evolution of applications of ctDNA in lymphomas.

Reference no.	Technique	Focus	No of patients	Key findings
**Diffuse Large B Cell Lymphoma**				
Kurtz et al. [20]	IgHTS	Baseline evaluation	75	Prognostic value of baseline ctDNA levels. Correlation of ctDNA with TMTV
Roschewski et al. [59]	IgHTS	Baseline and interim evaluation	126	Detectable interim ctDNA associated with risk of recurrence
Rivas-Delgado et al. [24]	Targeted NGS	Baseline evaluation	79	Higher ctDNA correlated with tumor burden. ctDNA determines tumor mutational profile and genetic classification.
Kurtz et al. [21]	CAPP-Seq	Baseline and dynamic assessment	217	Pretreatment ctDNA levels are associated with EFS and OS following front-line treatment A 2-log or 2.5-log fold decrease in ctDNA levels from baseline after 1 or 2 cycles of chemotherapy respectively is an independent biomarker of disease response.
Kurtz et al. [28]	PhasED-Seq	Dynamic assessment	213	ctDNA negativity by ultrasensitive PhasED-seq after 2 cycles is associated with a significantly lower risk of relapse
Alig S et al. [25]	CAPP-Seq	Baseline evaluation	267	Higher diagnostic ctDNA levels are associated with shorter EFS independent of IPI and diagnosis to treatment interval.
Meriranta et al. [22]	Targeted NGS (all); WGS (8 samples)	Baseline and dynamic assessment Fragmentation patterns	101	High pretreatment baseline ctDNA levels were associated with inferior OS. End of treatment ctDNA positivity (MRD) was associated with inferior OS
Roschewski et al. [29]	PhasED-Seq	Dynamic assessment	112	Detectable ctDNA at the end of treatment (MRD) is a strong predictor of relapse/refractory disease.
Frank et al. [32]	IgHTS	Treatment response prediction	72	Detectable ctDNA at day 28 following CD19 CAR-T (Axi-cel) was associated with median OS of 3 months versus not reached if undetectable.
Sworder et al. [34]	CAPP-Seq	Treatment response prediction	138	Both tumor effector T-cell cfDNA can be isolated from a single plasma sample to enable the evaluation of lymphoma and CAR-T cells
**Primary and secondary CNS lymphoma**				
Hattori et al. [38]	ddPCR Targeted NGS	Baseline and dynamic assessment	14	*MYD88* L265P is detectable by ddPCR in plasma cfDNA but only in just under 60% of the cohort.
Watanabe et al. [37]	ddPCR for *MYD88*	Baseline evaluation	26	*MYD88* driver mutations can be detected by a combination of Sanger sequencing and ddPCR in the CSF derived cfDNA.
Bobillo S et al. [39]	ddPCR, WES, and targeted NGS	Baseline and dynamic assessment	19	CSF ctDNA detection outperforms flow cytometry in the detection of MRD following treatment.
Mutter et al. [41]	CAPP Seq PhasED-Seq	Baseline and dynamic assessment	92	CtDNA is detectable by PhasED-Seq at all disease time points and is a robust biomarker predicting clinical outcome.
Heger et al. [42]	Targeted NGS	Baseline and dynamic assessment	67	Developed a risk stratification model for PCNSL using a combination of clinical and plasma-derived ctDNA features
**Hodgkins Lymphoma**				
Spina V et al. [43]	CAPP-Seq	Baseline and dynamic assessment	112	Clonal evolution can be tracked during and after treatment in longitudinal ctDNA samples
Alig et al. [44]	PhasED-Seq	Baseline and dynamic assessment	366	Pretreatment and on-treatment ctDNA levels are superior to conventional radiological assessment in MRD detection.
**Follicular Lymphoma**				
Sarkozy et al. [47]	IgHTS	Baseline evaluation	34	Clonal heterogeneity is detectable in ctDNA and ctDNA concentration at diagnosis has prognostic significance.
Fernández-Miranda et al. [49]	Targeted NGS	Baseline and Dynamic assessment	36	Failure to clear ctDNA during treatment is associated with poorer clinical outcomes including POD24
Jiménez-Ubieto et al. [50]	Targeted NGS	Dynamic assessment	84	The integration of ctDNA with radiological measures of disease burden allowed for a better assessment of disease burden in comparison to PET/CT alone.

TMTV, total metabolic tumor volume; IgHTS, immunoglobulin high-throughput sequencing; NGS, next-generation sequencing; EFS, event-free survival; OS, overall survival; IPI, international prognostic index; WGS, whole genome sequencing; MRD, measurable residual disease; PCNSL, primary CNS lymphoma.

#### Baseline

The early IgHTS-based study by Kurtz and colleagues demonstrated that baseline ctDNA abundance correlated with total metabolic tumor volume (TMTV) quantified by FDG-PET imaging [20]. The role of baseline ctDNA levels (derived by CAPP-seq methodologies) as a predictor of clinical outcome has been confirmed in several studies. One of the first and largest studies to date examined 217 DLBCL patients from 6 international institutions and showed that pretreatment baseline ctDNA levels were prognostic for event-free survival (EFS) and overall survival (OS), both following front-line treatment, as well as, pre-relapse treatment [21]. These were reaffirmed in clinical trials and real-world cohorts [22–24]. High pretreatment baseline ctDNA levels (defined as >3.75 log hGE/ml) in 101 high-risk DLBCLs from the Nordic LBC-05 Phase 2 trial (NCT01325194) were associated with inferior OS. Furthermore, these studies highlighted that higher baseline ctDNA burden remained a strong predictor of clinical outcomes on multivariate analyses and positively correlated with radiological and clinical determinants of high tumor burden such as TMTV, LDH, advanced stage, and higher international prognostic index (IPI) scores. Notably, high baseline ctDNA levels could also predict short diagnosis-to-treatment interval (DTI) emphasizing the valuable information it provides for early risk assessment and treatment planning, particularly for aggressive DLBCLs [21,22,24,25]. At baseline, one potential value of ctDNA analyses could be to capture the genetic profile of DLBCLs noninvasively using the blood ctDNA. Rivas-Delgado and colleagues found that the baseline ctDNA mutational profile could capture up to 70% of the tumor biopsy genetic profile, with just under 50% classified into the DLBCL molecular genetic subgroups using the LymphGen classifier. The lower percentage of patients classified in this study is most likely explained by the limited 112 gene panel used [24]. Nevertheless, the potential for upfront molecular subtyping from ctDNA would be invaluable in cases where the tumor biopsy is not amenable to molecular testing, for example, where the tissue is too small and/or poor quality.

#### Dynamic assessment: interim and end of treatment

As ctDNA levels change following treatment, in addition to baseline ctDNA levels, the role of the dynamics of ctDNA levels at interim treatment time points is also of interest to determine its value for predicting clinical outcomes and potential for treatment guidance. Kurtz and colleagues found that a 2-log or 2.5-log fold decrease in ctDNA levels from baseline after 1 or 2 cycles of chemotherapy, respectively, distinguished complete responders from non-responders and were predictive of EFS and OS [21]. Despite the prognostic accuracy of ctDNA levels at these fixed interim time points, the dynamics of ctDNA levels continue to temporally change and could contribute to later evolving risk in patients. To account for this, the authors developed a dynamic risk prediction algorithm called Continuous Individualized Risk Index (CIRI) that incorporated multiple pretreatment and during-treatment variables (including IPI, pretreatment and interim ctDNA levels, imaging) to increase the accuracy of the risk prediction [26]. The CIRI approach was a significantly superior outcome prediction tool compared to current conventional methods.

Traditionally, radiographic imaging by PET-CT is the current standard of care for response assessment and is prognostic at the end of front-line treatment (EOT). However, as high as 40% of DLBCL patients who are PET-positive at the EOT do not progress and equally, up to 20% who despite being PET-negative go on to have a progression event, illustrating the limitations in the sensitivity and specificity of imaging in detecting low-level residual disease [27]. As disease burden is lowest at EOT, highly sensitive ctDNA assays with a much lower limit of detection for residual disease such as PhasED-seq are needed to identify the most high-risk DLBCL patients. Kurtz and colleagues demonstrated that ctDNA negativity by ultrasensitive PhasED-seq after 2 cycles was associated with a significantly lower relapse risk, compared to CAPP-seq ctDNA negativity [28]. More recent studies have evaluated PhasED-seq at the end of the treatment time point. A pooled analysis by Roschewski and colleagues evaluated ctDNA MRD-negativity by phased-variants monitoring in 112 DLBCL patients across 5 trials treated with anthracycline-based curative-intent regimens. Persistently detectable ctDNA at EOT identified the highest risk group and EOT ctDNA MRD had a 94% sensitivity for identifying progression events. Building on this, Roschewski and colleagues conducted a study comparing the end-of-treatment response assessment using ctDNA analysis by PhasED-Seq versus a singular PET/CT scan assessment. Their data illustrated that ultrasensitive ctDNA EOT assays better predicted PFS than conventional imaging assessment PET-CT (HR: 36.3 vs 3.21). These suggest that the lack of specificity of conventional imaging and that PET positivity might lead to a significant number of patients who were destined not to progress being overtreated and raises the possibility of adopting EOT ctDNA MRD measurements to enhance our response criteria in DLBCL [29,30].

#### Response prediction for novel therapies

Cherng and colleagues reported on a Phase I/II study investigating the combination of lenalidomide and obinutuzumab with CHOP chemotherapy for newly diagnosed DLBCL patients and performed ctDNA genomic profiling using both CAPP-Seq and PhasED-Seq to track tumor dynamics and response to treatment. They demonstrated that this combination therapy led to a high rate of undetectable ctDNA after at least 5 cycles of therapy [31].

Recent studies have elucidated the pivotal role of ctDNA in monitoring treatment response and detecting early relapse post-infusion of chimeric antigen receptor T-cell (CAR-T cell) therapy. In a prospective multi-institutional trial, Frank and colleagues demonstrated that monitoring ctDNA levels significantly improves the early detection of relapse following axicabtagene ciloleucel infusion in DLBCL patients [32]. Another study demonstrated that integrating ctDNA analysis with PET imaging enhances the specificity of relapse detection. The authors demonstrated that PET-positive cases with no evidence of tumor-specific DNA are at a lower risk of disease progression and postulating that PET positivity in these cases is due to ongoing hyper-metabolic inflammatory response. These findings demonstrate that incorporating ctDNA and PET imaging can improve the overall efficacy of surveillance strategies post-CAR T-cell therapy [33].

ctDNA also offers insights into the mechanisms of disease resistance to CAR-T therapies. Sworder and colleagues investigated the utility of ctDNA analysis in investigating the determinants of resistance to CD19-targeted engineered T-cell therapies by an innovative simultaneous tumor and effector profiling (STEP) approach, which allows the capture of the tumor ctDNA and effector T-cell cfDNA from the same plasma sample to enable evaluation of both the lymphoma and the CAR-T cells. They identified genetic alterations in key genes involved in B-cell identity (*PAX5* and *IRF8*), immune checkpoints (*CD274*), and an immunosuppressive tumor-microenvironment in ctDNA samples. Additionally, they identified specific genomic alterations involved in immune evasion and apoptosis pathways which correlated to poor prognosis, treatment resistance, and relapse. A further study found that the changes in ctDNA levels during CAR-T treatment closely correlated to treatment response with rising ctDNA levels often preceding clinical and radiological evidence of relapse demonstrating the utility of dynamic ctDNA monitoring in this patient group [34,35]. These studies demonstrate that ctDNA can serve as a powerful tool to enhance the efficacy of CART-cell treatments in DLBCL by identifying resistance mechanisms, allowing for early intervention to prevent treatment failure.

### ctDNA studies in primary CNS lymphoma (PCNSL)

PCNSL represents a challenging diagnostic entity due to its rarity, aggressive disease course and difficulty in obtaining tissue biopsies from the central nervous system for analysis. CtDNA offers a promising noninvasive alternative to conventional high-risk invasive diagnostic approaches. Due to anatomical proximity, ctDNA can be more readily detected in CSF-derived cfDNA than plasma-derived cfDNA in CNS tumors.

Multiple studies have demonstrated the utility of ddPCR in detecting canonical recurrent point mutations, specifically *MYD88 L265P* (present in over 80% of PCNSL tumors) in CSF-derived ctDNA samples from primary and secondary CNS lymphoma patients [36]. Watanabe and colleagues used a ddPCR approach to demonstrate that *MYD88* L*265*P mutations were detectable in diagnostic CSF samples from 20/26 cases of CNS lymphoma [37]. The clinical utility of longitudinal *MYD88 L265P* mutation tracking in both plasma and CSF for risk stratification and disease monitoring has also been demonstrated in a number of studies [38–40]. Mutter and colleagues assessed ctDNA from CSF and plasma in 92 patients with PCNSL using CAPP-seq and PhasED-seq. They demonstrated baseline ctDNA burden is an independent predictor of prognosis and that clearance of ctDNA after 2 or 4 cycles of therapy was associated with significantly improved event-free survival [41]. They also demonstrated high specificity (97%) and sensitivity (100%) for the detection of ctDNA in CSF samples using the PhasED-seq platform, significantly outperforming radiological modalities of response assessment and other less sensitive ctDNA assays. More recently, Heger and colleagues performed ultrasensitive ctDNA sequencing on plasma and CSF samples from 67 patients in a focused investigation aimed at outcome prediction in CNS lymphomas. They identified distinct molecular signatures associated with disease progression and response to therapy, demonstrating that ctDNA profiling has utility in prognostication as shown in Table 1 [42].

### ctDNA studies in Hodgkin lymphoma (HL)

Classical Hodgkin lymphoma patients have a significantly higher burden of plasma ctDNA compared to other lymphoma subtypes. There is particular interest in ctDNA analysis in cHL owing to the scarcity of tumor cells and hence tumor DNA in tumor biopsy samples (Table 1). A study by Spina and colleagues demonstrated that mutation profiling of ctDNA complements traditional diagnostic methods by identifying somatic mutations and genetic alterations characteristic of cHL. Genomic profiling of ctDNA allows for a comprehensive understanding of disease biology [43]. Alig and colleagues used single-cell transcriptomics and PhasED-Seq to define distinct molecular subtypes of HL and were able to determine the underlying molecular heterogeneity which not only enhances the diagnostic accuracy but also offers the implications for prognosis and treatment response prediction. They demonstrated significant clinical value in pretreatment and on-treatment ctDNA levels for cHL risk prediction and superior sensitivity of ctDNA-based MRD detection methods compared to conventional radiological assessment [44]. Another study also demonstrated the superior prognostication of baseline ctDNA assessment using CAPP-Seq in addition to interim positron emission tomography (iPET) scans in relapsed/refractory cHL compared to radiological prognostication [45].

Another study using a ctDNA-targeted genotyping approach to monitor cHL patients during treatment failure to clear ctDNA during treatment predicted relapse earlier than the conventional imaging techniques. In a study by Lynch and colleagues investigated the efficacy of combining pembrolizumab with AVD chemotherapy in untreated cHL patients, clearance of ctDNA after 2 cycles of treatment and at the end of treatment were both associated with superior PFS even among the small group of patients with PET-positive findings at the end of treatment. These data indicate that ctDNA response assessment techniques can outperform conventional radiological methods and that dynamic ctDNA assessment is a strong predictor of treatment response, even at an early timepoint assessment, allowing for early treatment intensification for those patients with refractory disease [46,47].

### ctDNA studies in follicular lymphoma (FL)

Follicular lymphoma (FL) is the most common indolent NHL with heterogeneity in clinical behavior. There is a need to identify the proportion of patients who undergo early relapse or transformation to high-grade disease in order to tailor more intensive treatment regimens for this at-risk group.

The PRIMA trial included an assessment of ctDNA from 34 patients using clonoSEQ and demonstrated that tumor-specific VDJ clonotypes could be identified in the plasma ctDNA in 74% of cases. Higher baseline ctDNA levels were associated with significantly shorter PFS [48]. More recently, Fernández-Miranda and colleagues conducted a prospective pilot study using targeted sequencing of plasma-derived ctDNA from 36 patients with FL. They demonstrated concordance between mutations identified in ctDNA and in diagnostic tumor biopsies and that higher pretreatment ctDNA levels were associated with patients not achieving a complete response (CR) and in those with early progression within 24 months (POD24). Furthermore, interim ctDNA levels were most reduced in those who achieved a CR and those without POD24. This real-time tracking capability underscores the potential of ctDNA as a dynamic biomarker treatment response monitoring in FL [49].

Another study performed targeted sequencing of plasma ctDNA in conjunction with PET/CT in a cohort of 38 FL patients to provide a framework for disease monitoring. The integration of ctDNA allowed for a better assessment of disease burden in comparison to PET/CT alone and enhanced the ability to risk-stratify patients in order to potentially offer tailored treatment strategies [50]. Zhao and colleagues expanded on these findings, performing targeted sequencing on plasma-derived ctDNA in a cohort of 28 FL. They concluded that higher baseline ctDNA was predictive of inferior OS and PFS and demonstrated that the changes in ctDNA can serve as an early indicator of progression and treatment response [51].

### ctDNA studies in other lymphomas

#### Mantle cell lymphoma (MCL)

The role of ctDNA in response assessment for MCL has earned significant attention due to its potential to improve prognostic accuracy and guide therapeutic decisions. In one study by Lakhotia and colleagues, ctDNA was used to monitor therapeutic outcomes following induction therapy using PCR amplification of immunoglobulin heavy chain gene followed by NGS. The absence of detectable ctDNA after two cycles of chemotherapy was associated with longer PFS and OS. They also highlighted the role of ctDNA’s potential to guide preemptive therapeutic strategies and tailor treatment intensity based on the MRD status at the end of therapy and the early relapse and proved its superiority above the radiological methods in monitoring response and relapse. They illustrated that ctDNA can be used as a tool for monitoring clonal evolution that can lead to treatment resistance. This is particularly valuable in a heterogeneous disease like MCL, where individual responses to treatment can vary widely [52].

#### Marginal zone lymphoma (MZL)

In a study by Tatarczuch and colleagues, the authors used whole exome sequencing (WES) and targeted sequencing of ctDNA to uncover molecular determinants associated with response and resistance to zanubrutinib. The authors were able to detect mutations in key BCR signaling pathways (*NF-KB, NOTCH*, and *BCR)* associated with MZL in diagnostic plasma ctDNA samples. They were able to determine that *MYD88* or *TNFAIP3* variants were associated with longer PFS while *KMT2D* variants were associated with shorter PFS. The authors also identified mutations in *PLCG2* and *BTK* acquired during zanubrutinib therapy which were responsible for BTKi resistance and associated with on-study disease progression. The ability to track molecular changes in ctDNA in real-time allows for more responsive and adaptive treatment strategies, potentially improving patient outcomes in relapsed or refractory MZL [53].

#### T-cell lymphomas

CtDNA analyses have been less well studied in T-cell lymphomas, likely due to their rarity and difficulty in assembling large cohorts for analysis. Despite this, there is interest in ctDNA T-cell lymphomas as clinical outcomes remain significantly worse than in B-cell lymphomas. Wei and colleagues performed targeted sequencing on a cohort of 36 peripheral T-cell lymphoma (PTCL) patients. They were able to perform comprehensive mutational profiling from ctDNA samples alone, sufficient to distinguish different subtypes of PTCL with high fidelity compared to tissue biopsies. They highlighted that specific genetic mutations detected through ctDNA analysis were associated with inferior prognostic outcomes. The authors also demonstrate that, as in B-cell lymphomas, baseline plasma ctDNA concentration is significantly correlated to radiological measures of tumor burden and that ctDNA profiling allowed for risk stratification and the potential to make early clinical decisions regarding treatment intensification [54].

## Novel approaches to ctDNA analysis

In addition to genomic profiling of ctDNA for genetic alterations and copy number changes, methylation profiling of ctDNA has emerged as a transformative approach for noninvasive cancer diagnostics, prognostics, and monitoring of cancers. Genome-wide epigenetic alterations are tissue and cancer-type specific and the detection of these methylation signatures can be leveraged in the development of ctDNA assays. Shen and colleagues performed genome-wide methylation profiling (using cell-free methylated DNA immunoprecipitation and high-throughput sequencing, cfMeDIP–seq, on plasma-derived ctDNA from a range of tumor subtypes and demonstrated sensitive tumor detection and tumor subtype classification across various cancer types. This research paved the way for the development of a comprehensive diagnostic platform using methylation patterns [55]. Zuccato and colleagues developed a novel approach using CSF methylome-based liquid biopsies for accurate classification of malignant brain neoplasms using cfMeDIP–seq. They were able to accurately distinguish CNS lymphomas from glioblastomas. There is considerable interest in this kind of ctDNA assay as it obviates the need for an invasive, high-risk CNS tissue biopsy [56].

Additionally, the study conducted by Tian and colleagues investigated the potential role of ctDNA methylation patterns in extranodal natural killer/T cell lymphoma (ENKTL). The authors identified distinct ctDNA methylation signatures associated with ENKTL. The presence of these methylation signatures was associated with adverse clinical prognosis suggesting their potential utility in guiding treatment decisions and monitoring disease progression [57].

Investigating cfDNA and ctDNA fragmentation patterns is also of growing interest. In a study from 101 DLBCL patients, it was demonstrated that plasma ctDNA from these patients, had a distinct fragmentation pattern compared to healthy cfDNA and a machine learning strategy was leveraged to distinguish between ctDNA versus cfDNA. The presence of distinct end-of-treatment ctDNA fragmentomic patterns were predictive of adverse patient outcomes and with that the possibility of an alternative to mutation-based MRD detection [22].

Esfahani and colleagues recently published their novel approach to infer gene expression from cell-free DNA fragmentation profiles using “epigenetic expression inference from cell-free DNA-sequencing” (EPIC-seq). In both non-small lung cancer and DLBCL cohorts, epigenetic profiling from ctDNA could be used to correctly subtype tumors and inform treatment response [58].

## Bringing ctDNA analysis into routine clinical practice

The clinical validity of using ctDNA analyses as a biomarker has been confirmed with many recent studies for lymphoma detection, disease monitoring, and prognostication, particularly in DLBCL. It is important to highlight that the place and value of ctDNA analyses differ across the lymphoma subtypes. For example, lymphoma subtypes where the goal of treatment is curative such as DLBCL and HL, the laser focus will be using ctDNA analyses to identify those non-responders to conventional therapy early so as to divert them toward alternative curative options; whereas primarily non-curative indolent lymphomas, ctDNA analyses might be more beneficial to guide the need for maintenance or consolidation therapies. However, there remain some critical gaps that hinder its adoption into routine clinical practice. Firstly, there currently are an array of technical options for ctDNA assays with wide-ranging sensitivities. The required or optimum assay sensitivity will have suitability for different clinical applications, for example a less sensitive assay could be used for diagnostic testing however, a much higher assay sensitivity will be needed for minimal residual disease detection or in early-stage disease where tumor burden may be low. Improving the sensitivity requires advances in both sequencing technologies and bioinformatics algorithms to accurately detect and quantify minute ctDNA fractions amidst background noise. In addition to analytics, standardized protocols for sample collection and processing are imperative to ensure reproducibility and comparability across studies and clinical settings. Altogether, these have implications for the analytical validity required for clinical implementation, in terms of the accuracy, robustness and reproducibility of these assays. This intuitively also needs to be balanced with the healthcare cost feasibility and ease of implementation into routine clinical workflows. Lastly, to confirm the clinical utility of ctDNA-based biomarkers, large-scale prospective clinical trials are warranted across the lymphomas assessing their performance in prognostication, treatment response monitoring, and early detection of relapse. Indeed, it is very pleasing that several of these are ongoing across the major lymphoma subtypes. Bridging these gaps will be instrumental in realizing the full potential of ctDNA as a valuable tool in the clinical management of lymphoma.

## Conclusion

Liquid biopsy techniques, particularly those analyzing circulating tumor DNA (ctDNA), have shown significant promise as a noninvasive alternative to traditional tissue biopsies for lymphoma diagnosis, monitoring, and relapse detection. Recent advances in next-generation sequencing and machine learning have enhanced the sensitivity and specificity of ctDNA assays. These techniques offer valuable insights into tumor dynamics, treatment response, and disease progression across various lymphoma subtypes, including DLBCL, PCNSL, HL, FL, and MCL. Despite their potential, challenges such as optimizing sampling techniques and assay sensitivity remain. Continued research and technological improvements are needed to fully integrate ctDNA assays into routine clinical practice for lymphoma management.
